# Appendicitis with direct fistulation into the liver: a forgotten cause of pyogenic liver abscess

**DOI:** 10.1259/bjrcr.20200101

**Published:** 2020-08-07

**Authors:** Tom Armstrong, Sam Dluzewski, Dominic Yu

**Affiliations:** 1 Radiology Registrar, Royal Free Hospital, London, UK; 2 Consultant Interventional & Hepatobiliary Radiologist, Royal Free Hospital, London, UK

## Abstract

Pyogenic liver abscess typically occurs secondary to biliary or haematogenous spread of organisms. In the context of acute appendicitis, abscesses generally occur due to haematogenous spread through the mesenteric vasculature. Historically, few cases of direct intra-abdominal spread have been reported but this has become vanishingly rare since the development of antibiotic therapy with no recorded cases in a search of over 900 cases in the literature.

We present a case of a 69-year-old female patient who presented with fever and shortness of breath who was subsequently diagnosed with a pyogenic liver abscess and empyema due to perforated appendicitis with direct fistulation. The initial diagnosis was made on chest radiograph (Figure 1). This led to timely optimal treatment of the patient including percutaneous drainage of the liver abscess and empyema.^1–3^

**Figure 1.**
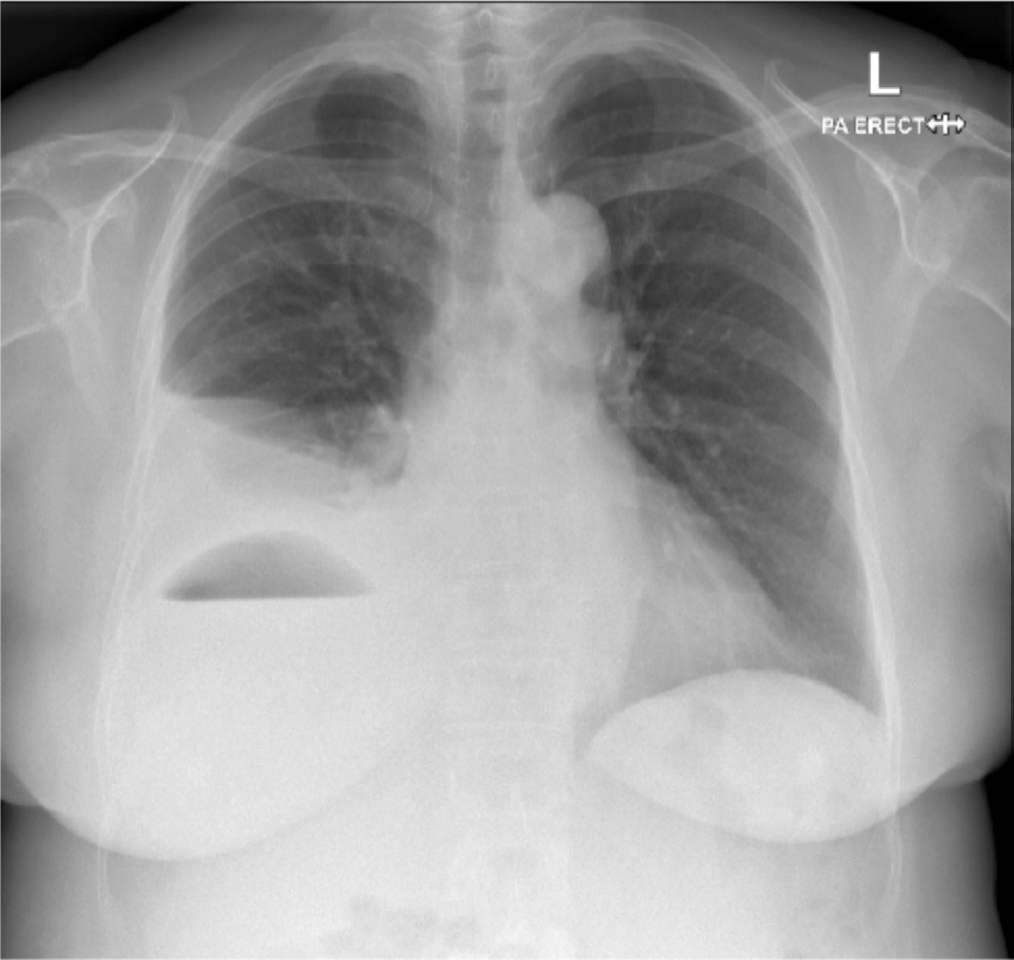
PA chest radiograph taken at presentation demonstrating a right-sided pleural effusion tracking in the oblique and horizontal fissures as well as a sub-diaphragmatic collection with air-fluid level. Note the superior margin of the air crescent following the perceived diaphragmatic contour which alludes to this being abdominal in location.

This case report demonstrates the importance of thorough radiological investigation in the context of suspected hepatic abscess in order to delineate important albeit rare causes that may not resolve with conservative management alone.

## Introduction

Appendicitis is not only a common surgical emergency but also a frequent indication for CT examination of the abdomen and pelvis. There is an approximate appendix perforation rate of 10–20% which can depend on different factors.^4^ The overwhelming majority of perforations occur intraperitoneally with an inflammatory fluid collection or phlegmon forming around the inflamed vermiform appendix.

Non-specific but important sequelae of acute appendicitis are associated with single or multiple pyogenic liver abscesses. This classically occurs due to haematogenous spread of bowel organisms via the mesenteric venous drainage entering the portal venous system and subsequent seeding into the liver. The other classical common cause of pyogenic abscess would be due to the biliary spread of organisms, for example cholecystitis or ascending cholangitis.^5^

The third route of spread is direct seeding of organisms into the liver. This mostly occurs via direct iatrogenic inoculation following a procedure (*e.g.,* biopsy) or some form of penetrating trauma. We present an extremely uncommon case of contiguous pyogenic liver abscess in the context of a subacute/missed appendicitis that has become adherent to and subsequently fistulated into the right lobe of the liver.

## Case presentation

A 69-year-old female patient presented to the emergency department following a several week history of malaise, shortness of breath and fever with an insidious onset. There was a history of some foreign travel to Singapore 12 months prior. On closer questioning, she had also experienced intermittent nausea,vomiting and diarrhoea. She denied any history of preceding abdominal pain. She was an ex-smoker with a background of hypertension for which she takes amlodipine and mild asthma with PRN inhalers. On arrival to the emergency department, the patient was afebrile (36.7°C), slightly hypertensive at 153/90 mm Hg and with a respiratory rate of 18/min and an oxygen saturation of 96% on room air. Serum blood markers identified a neutrophilia of 14.1 (10^9^/L), an elevated serum lactate of 2.8 (mg/dL) and a C-reactive protein of 221 (mg/L). An admission radiograph demonstrated complex features with a large right-sided pleural effusion and a gas-fluid level that “mirrored” the diaphragmatic contour raising the suspicion for a hepatic or subdiaphragmatic abscess (Figure 1). A bedside ultrasound was performed in the emergency department which suggested the pleural fluid was anechoic and simple on admission.

**Figure 2.**
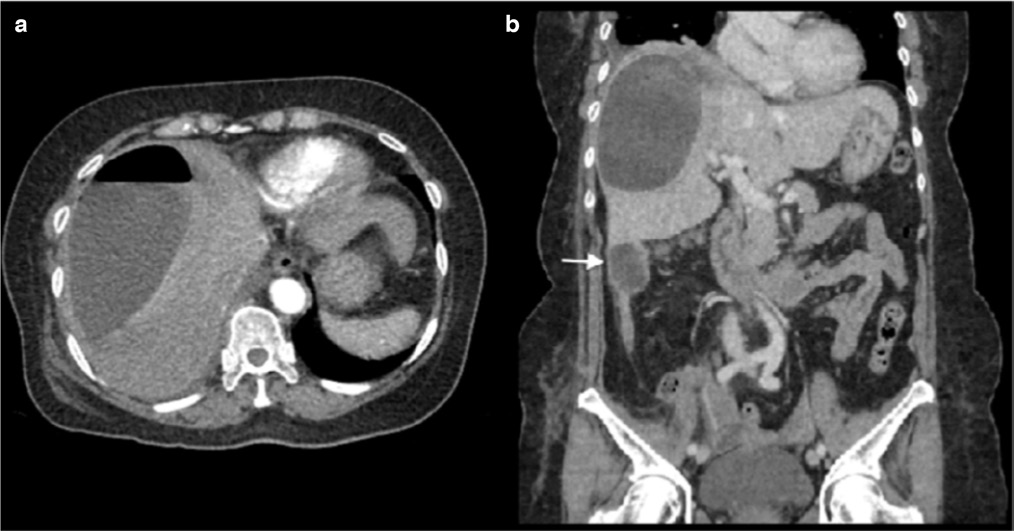
(A) Arterial axial CT scan of the upper abdomen demonstrating a large gas/fluid abscess within the right lobe of the liver (patient lyingsupine). (B) Coronal CT scan with portal venous contrast. Note the expanded perforated fluid collection arising from the tip of the appendix (white arrow) that is contiguous with the inferior liver margin.

**Figure 3.**
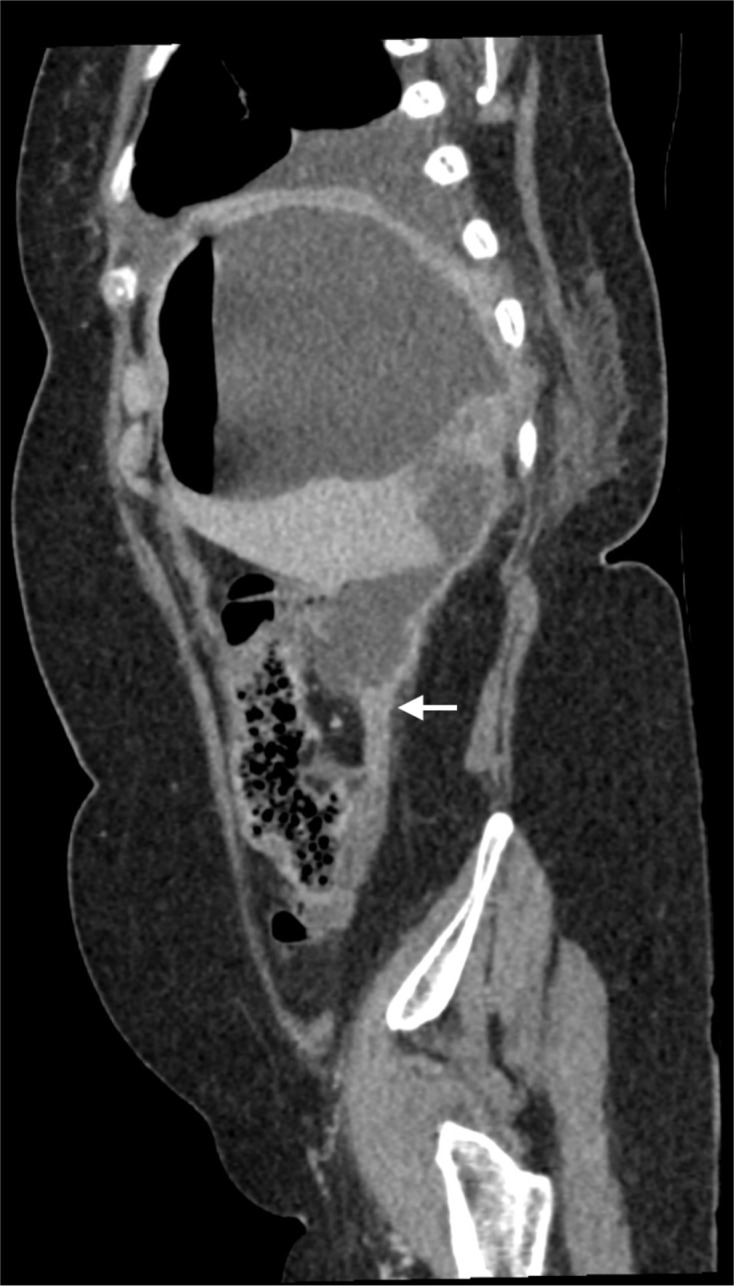
Sagittal CT scan with reconstruction to demonstrate the contiguous nature of the large pyogenic abscess extending from the right hepatic lobe inferiorly through multiple lobulated interconnected fluid collections which can be seen arising from the appendiceal tip (white arrow) in turn arising from the retrocaecum.

**Figure 4.**
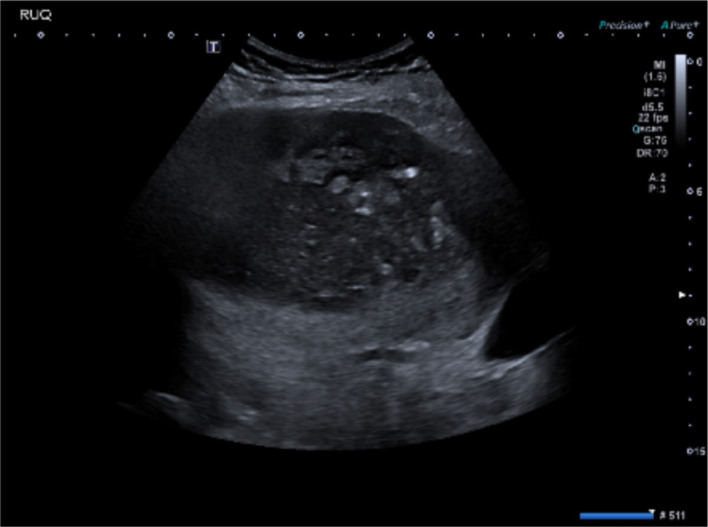
Ultrasound scan of right hepatic lobe gas containing heterogeneous mixed echogenicity lesion centrally within the right lobe. The hyperechoic foci likely represent trapped gas locules.

Following discussion with the on-call radiologist, a CT scan of the abdomen and pelvis was performed with triple-phase imaging of the liver to accurately localize and characterize the plain film abnormality. The scan confirmed a large right hepatic lobe subcapsular abscess however it demonstrated a rare and unusual aetiology. The appendix was noted to be distended with evidence of perforation of the tip which had become adherent to segment VI of the inferior margin of the right lobe of the liver. This has then led to contiguous intrahepatic perforation via a fistulating tract (Figures 2 and 3). Following this a diagnostic ultrasound was performed in the radiology department to characterize the contents of the abscess and determine whether it is amenable to percutaneous drainage (Figure 4).

**Figure 5.**
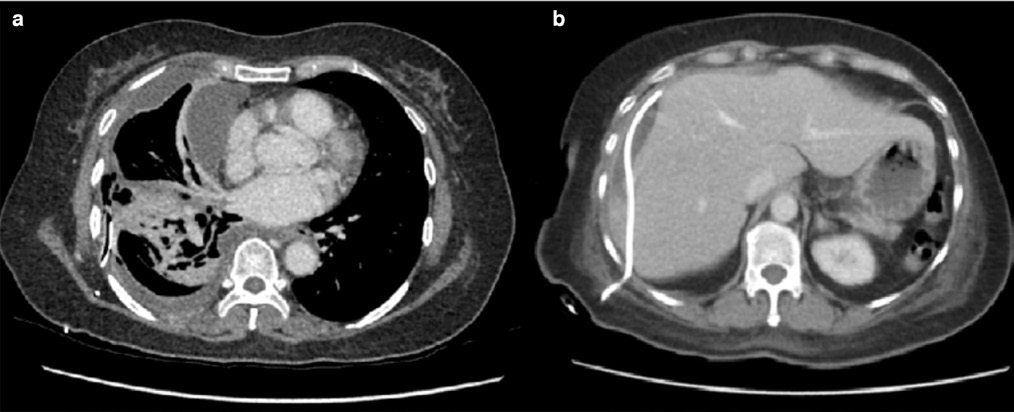
(A) Axial CT scan with portal venous phase contrast of the thorax demonstrating a right sided empyema. Note the enhancing pleural surfaces. A right-sided intercostal drain is seen. (B) Unenhanced MIP’d image of the upper abdomen demonstrating the right-sided intercostal pigtail hepatic drain with marked improvement in the appearances of the abscess.

Ultrasound-guided percutaneous drainage of the subcapsular liver abscess was performed two days following initial presentation within the interventional radiology department. An 8.5Fr locking pigtail drain was placed into the largest fluid component of the abscess and 550 ml of purulent fluid was aspirated and cultures grew gram-negative rods and cocci. Blood cultures demonstrated no growth. A right-sided pleural drain was inserted on the ward due to the reactive right-sided effusion and supposed possibility of contiguous spread^6^ (Figure 5). The patient was commenced on i.v. antibiotics and was discharged following oral conversion to complete her course at home with her right pleural drain being removed prior to discharge.

**Figure 6.**
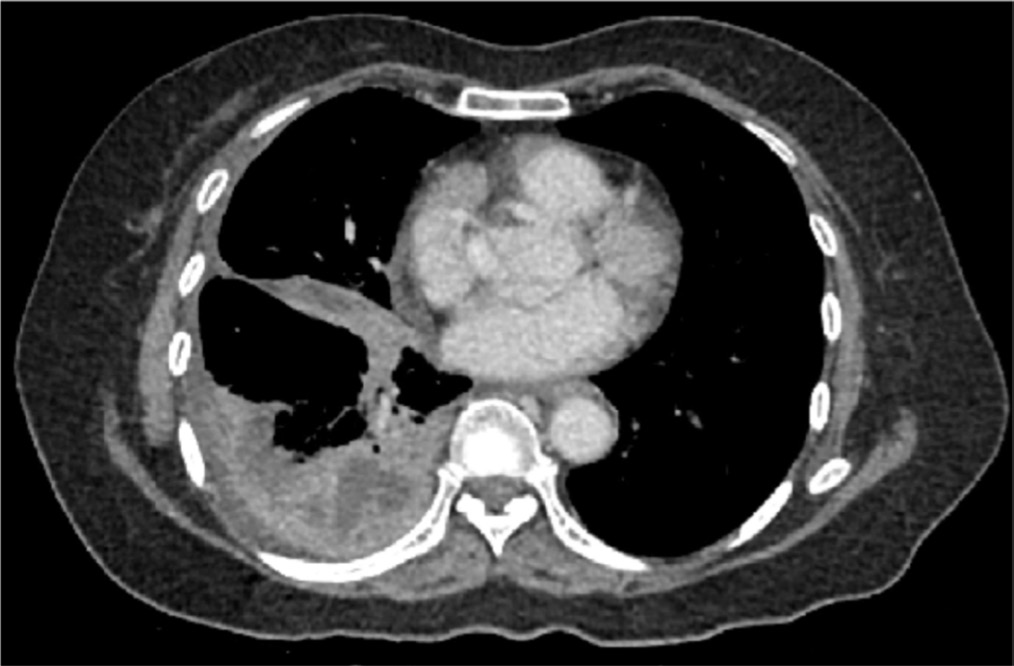
Axial CT scan with contrast of the lower thorax on re-admission.The right lower lobe parenchyma demonstrates central low density geographic fluid collections raising the in keeping with secondary necrotizing pneumonia associated with the empyema.

The patient subsequently represented to Accident and Emergency 48 h following discharge with a new temperature, malaise and increasing right thoracic/upper abdominal pain. Clinical observation and biochemical analysis revealed a systemic inflammatory response syndrome and she was re-admitted. Repeated blood cultures remained negative. A repeat CT scan was performed and while this did show persistent albeit improving abscess in the liver, the thoracic findings had progressed. The scan demonstrated right lower lobe necrotizing pneumonia and ongoing evidence of pleural enhancement in keeping with the known empyema (Figure 6). A right-sided pleural drain was re-inserted and culture of the aspirate grew gram-negative rods and cocci. Following repeated antibiotic treatment, the patient was discharged for outpatient follow-up with consideration for appendectomy as an outpatient.

## Discussion

As stated in the introduction, pyogenic liver abscess, which are often multiple, are characterized by pus containing poly-microbial collections and are most commonly caused by haematogenous spread from the gastrointestinal tract or ascending biliary tract infection or cholecystitis. Direct intraperitoneal extension from an intra-abdominal collection was historically identified as a recognized cause^1^ in the pre-antibiotic era which is now so uncommon in the modern healthcare setting that it is virtually undescribed in a retrospective case series review.

Management considerations are usually based upon an appraisal of the number and size of the abscesses where smaller lesions, particularly those under 50 mm in size, may be amenable to medical management.^7^ Given these important management implications, radiology plays a central role in the assessment of hepatic abscess(s). While simple bedside tests may allude the clinician to the possibility of an abscess, CT remains an important modality for a full assessment of not only the size and number of lesions but as demonstrated in this case, the potential aetiology. Direct spread from intra-abdominal infection can be an indicator for required interventional or surgical treatment in combination with antibiotic therapy.^3^ Management includes consideration for appendicectomy to reduce the risk of continued bowel contents entering the liver/pleural space despite optimal drainage as is likely in the case we describe.

In the context of a pyogenic liver abscess, a patient would typically present with fever and right upper quadrant pain in greater than 60% of cases. This was not the case with our patient and this atypical presentation can require quick recognition of abnormal radiological findings in the emergency setting to guide appropriate further investigation and subsequent management. It is also worth noting that our case actually demonstrated a subcapsular abscess with two small intra-parenchymal breaches. This is even more unusual for the abscess to be tracking in the subcapsular space. Having an awareness of this pathology can help both clinicians and radiologists to think laterally and ensure they work together to investigate appropriately.

While appendicitis can occur at any age, given our patient’s age of 69 years and the atypical presentation of appendicitis in this case, for example insidious onset symptoms with no abdominal pain other considerations must be taken as to the underlying aetiology of the appendicular perforation. Acute/subacute inflammatory appendicitis is a very common pathology and while this still remains the most likely aetiology radiologically, one must consider rarer causes such as an appendiceal tumour. While it is known that an appendicular malignancy can mimic acute appendicitis, it may also have a more insidious onset and lack the typical clinical features of a benign inflammatory appendicitis.^8^ Appendicular malignancy is found in around 1% of all post-mortem appendix specimens.^9^ Epithelial neoplasms (mucinous tumours of the appendix) and neuroendocrine tumours (carcinoid tumours) account for the vast majority. It is worth noting that in our case, there was no evidence of pseudomyxoma peritoneii which can be seen on CT when mucinous tumours of the appendix rupture. Interestingly carcinoid tumours of the appendix are seen within the tip in 75% of cases.^10^

This case highlights that one must always consider common conditions presenting atypically and the importance of appropriate and timely radiological investigation in such cases. In the context of suspected hepatic abscess, this helps to localize pathology, assess for some key aetiologies and detect worrying sequelae as demonstrated in this case. This case highlights the importance of increasing both clinicians and diagnostic radiologists awareness of this now rare pathology to ensure timely diagnosis and intervention. This case also offers an opportunity to revisit the pathology of the appendix and consider both benign inflammatory and neoplastic causes of appendiceal perforation.

## Learning points

1. Radiologists should consider direct intraperitoneal spread as a cause of pyogenic liver abscess.
2. Have a low threshold for cross-sectional imaging in assessing suspected pyogenic liver abscess.
3. Ultrasound can help to guide management in planning percutaneous drainage of intra-hepatic abscess.
4. In older populations, one must consider differential diagnoses of appendiceal perforation such as an appendiceal tumour.

## References

[1] Ochsner A, DeBakey M, Murray S Pyogenic abscess of the liver. The American Journal of Surgery 1938; 40: 292–319. doi: 10.1016/S0002-9610(38)90618-X

[2] Serraino C, Elia C, Bracco C, Rinaldi G, Pomero F, Silvestri A, et al Characteristics and management of pyogenic liver abscess: a European experience. Medicine 2018; 97: e0628. doi: 10.1097/MD.0000000000010628 29742700 PMC5959441

[3] Mangukiya DO, Darshan JR, Kanani VK, Gupta ST A prospective series case study of pyogenic liver abscess: recent trands in etiology and management. Indian J Surg 2012; 74: 385–90. doi: 10.1007/s12262-011-0397-0 24082591 PMC3477416

[4] Andersson RE, Hugander A, Thulin AJ Diagnostic accuracy and perforation rate in appendicitis: association with age and sex of the patient and with appendicectomy rate. Eur J Surg 1992; 158: 37–41. 1348639

[5] Bortoff GA, Chen MY, Ott DJ, Wolfman NT, Routh WD Gallbladder stones: imaging and intervention. Radiographics 2000; 20: 751–66. doi: 10.1148/radiographics.20.3.g00ma16751 10835126

[6] May J, Ades A Porous diaphragm syndrome: haemothorax secondary to haemoperitoneum following laparoscopic hysterectomy. BMJ Case Rep 2013; 2013: bcr201320108805 Dec 20132013(dec05 2):bcr2013201088-bcr2013201088. doi: 10.1136/bcr-2013-201088 PMC3863045 24311458

[7] Tan Y-M, Chung AY-F, Chow PK-H, Cheow P-C, Wong W-K, Ooi LL, et al An appraisal of surgical and percutaneous drainage for pyogenic liver abscesses larger than 5 cm. Ann Surg 2005; 241: 485–90. doi: 10.1097/01.sla.0000154265.14006.47 15729072 PMC1356988

[8] Pickhardt PJ, Levy AD, Rohrmann CA, Kende AI Primary neoplasms of the appendix manifesting as acute appendicitis: CT findings with pathologic comparison. Radiology 2002; 224: 775–81. doi: 10.1148/radiol.2243011545 12202713

[9] Leonards LM, Pahwa A, Patel MK, Petersen J, Nguyen MJ, Jude CM Neoplasms of the appendix: pictorial review with clinical and pathologic correlation. Radiographics 2017; 37: 1059–83. doi: 10.1148/rg.2017160150 28598731

[10] Levy AD, Sobin LH From the Archives of the AFIP: gastrointestinal carcinoids: imaging features with clinicopathologic comparison. Radiographics 2007; 27: 237–57. doi: 10.1148/rg.271065169 17235010

